# Large Hall Signal due to Electrical Switching of an Antiferromagnetic Weyl Semimetal State

**DOI:** 10.1002/smsc.202000025

**Published:** 2021-04-15

**Authors:** Hanshen Tsai, Tomoya Higo, Kouta Kondou, Shoya Sakamoto, Ayuko Kobayashi, Takumi Matsuo, Shinji Miwa, Yoshichika Otani, Satoru Nakatsuji

**Affiliations:** ^1^ Institute for Solid State Physics University of Tokyo Kashiwa Chiba 277-8581 Japan; ^2^ CREST Japan Science and Technology Agency Kawaguchi Saitama 332-0012 Japan; ^3^ RIKEN Center for Emergent Matter Science (CEMS) Wako Saitama 351-0198 Japan; ^4^ Trans-scale Quantum Science Institute University of Tokyo Bunkyo-ku Tokyo 113-0033 Japan; ^5^ Department of Physics University of Tokyo Bunkyo-ku Tokyo 113-0033 Japan

**Keywords:** anomalous Hall effect, antiferromagnetic memory, spin–orbit torque, spintronics, Weyl semimetal

## Abstract

Developing a technology to electrically manipulate a Weyl semimetal state is a vital step for designing a nonvolatile memory using topologically robust properties. Recently, such manipulation is realized for the first time in the antiferromagnetic Weyl semimetal Mn_3_Sn using the readout signal of anomalous Hall effect in the Mn_3_Sn/heavy metal (Pt, W) heterostructures. Here, it is reported that the switching of Hall signal can be significantly enhanced by 1) removing the buffer layer of Ru to adjust the crystal orientation of Mn_3_Sn, and 2) annealing after deposition of the heavy metal to change the interfacial condition. The switching of the Hall resistance is 0.35 Ω in the Mn_3_Sn/W sample, which becomes one order of magnitude larger than the previously reported value using Ru/Mn_3_Sn/Pt heterostructures. Moreover, by increasing the read current, it is found that the readout voltage may go well beyond 1 mV, a milestone for future applications in memory technology.

## Graphical abstract

Antiferromagnets attract tremendous interest for spintronics application due to their small stray fields and fast dynamics. Antiferromagnetic Weyl semimetal Mn_3_Sn/heavy metal heterostructures can act as nonvolatile memory where the readout Hall voltage can be switched electrically. This study reports that this readout signal can be significantly enhanced to the order of millivolts by adjusting their crystal orientation and interfacial condition.

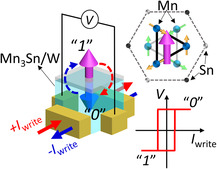

## 1 Introduction

In nanoscale technology, topologically protected properties are expected to take on a significant role in developing next generation electronic devices. Recent extensive studies indicate that such robust properties can be found in topological Weyl semimetals. Characterized by topologically protected Weyl points, acting as a source and drain of Berry curvature, the magnetic Weyl semimetals may exhibit large responses, such as anomalous Hall effect (AHE), anomalous Nernst effect (ANE), and chiral anomaly.^[^ ^1^, ^2^, ^3^, ^4^, ^5^, ^6^, ^7^, ^8^, ^9^, ^10^, ^11^, ^12^, ^13^, ^14^, ^15^, ^16^, ^17^, ^18^, ^19^, ^20^, ^21^, ^22^ ^]^ Developing the technology to electrically manipulate the topological responses should enable storing information by taking advantage of the robust topological properties, and, thus, designing a new type of nonvolatile memory.

**Figure 1:**
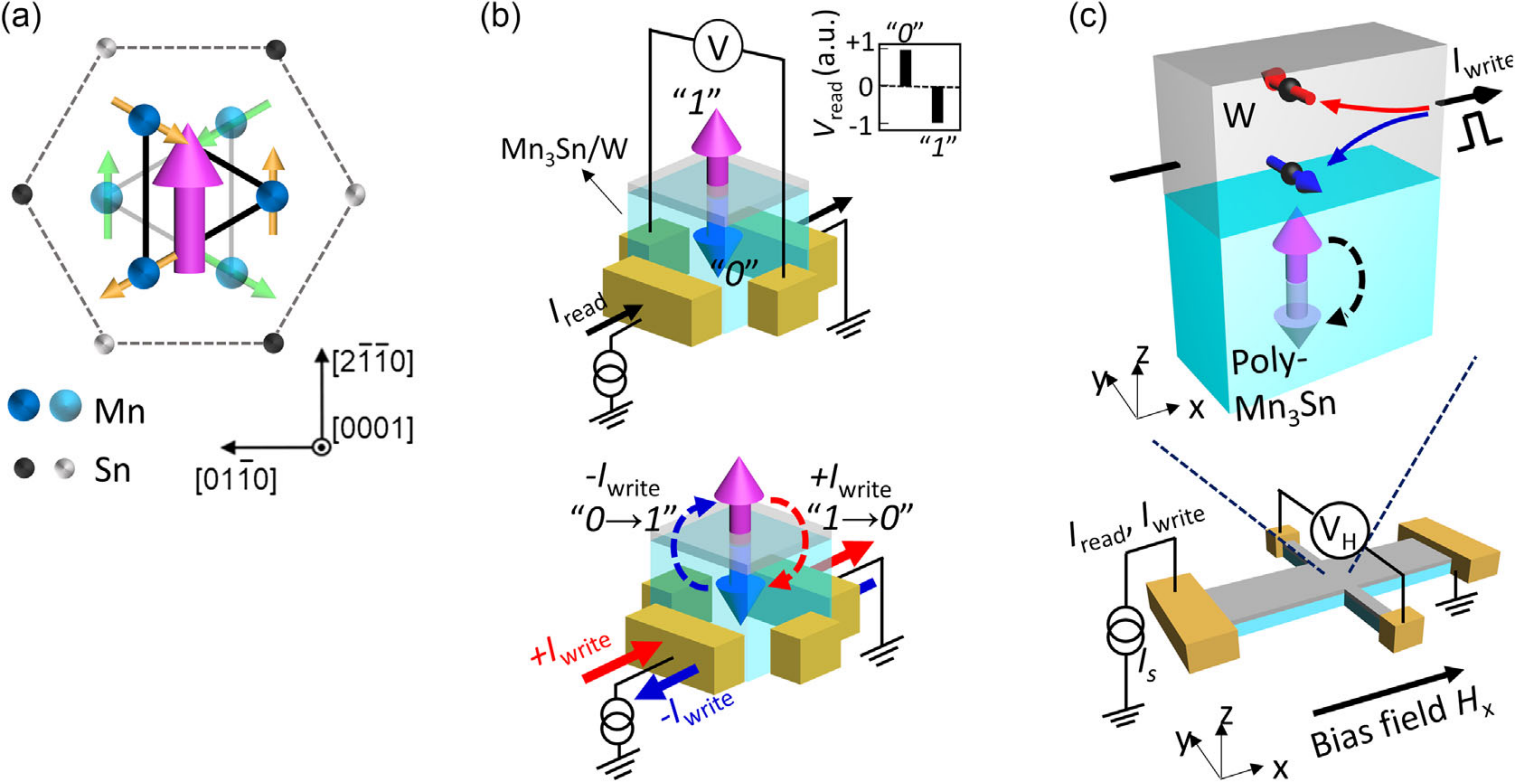
a) Crystal and spin structures of Mn_3_Sn. Blue and light blue spheres represent Mn atoms on the kagome bilayers, forming an octahedron. Black and gray spheres represent Sn atoms. The non‐collinear spin structure consisting six Mn magnetic moments (yellow and green arrows) can be viewed as ferroic ordering of a magnetic octupole (purple arrow). b) Schematics of a four‐terminal antiferromagnetic memory device based on Mn_3_Sn/W bilayer. Top: Read operation in memory. The sign of readout voltage is determined by the out‐of‐plane component in the polarization direction of the magnetic octupole of Mn_3_Sn (purple and blue arrows), namely, “0” or “1”state. Bottom: Write operation in memory. The switching direction between two states, “0→1” or “1→0,” is determined by the sign/direction of the write current *I*_write_. c) Bottom: Schematic image of Mn_3_Sn/heavy metal devices and the measurement setup of electrodes. The samples are fabricated in 16 μm × 96 μm Hall bar structure and contacted with Au/Ti electrodes. Top: Schematic image of the SOT switching in Mn_3_Sn/W bilayer. An electrical current flowing in W layer generates a spin current by the spin Hall effect of W. This spin current induces an SOT on Mn_3_Sn layer and causes the switching of the polarization direction of the magnetic octupole. Here, red and blue thin arrows represent the spin moments of the spin current. Purple and light purple thick arrows represent the polarization direction of the magnetic octupole.

Such electrical manipulation of large topological responses has been recently demonstrated for a magnetic Weyl semimetal. In particular, magnetic switching of the antiferromagnet Mn_3_Sn has been performed using spin–orbit torque (SOT) by applying electrical current in Ru/Mn_3_Sn/Pt or W heterostructure.^[^ ^23^ ^]^ Mn_3_Sn is a magnetic Weyl semimetal that exhibits large AHE and ANE at room temperature.^[^ ^9^, ^12^, ^13^, ^14^ ^]^ It is a metallic antiferromagnet with the hexagonal D0_19_ structure, having the ABAB stacking of the kagome lattice of Mn along [0001]. The geometrical frustration leads to 120° structure made of Mn magnetic moments with a negative vector chirality (**Figure** **1**a). Significantly, this non‐collinear structure can be viewed as a ferroic order of a cluster magnetic octupole^[^ ^24^ ^]^ made of six neighboring Mn moments. As this ferroic order breaks the time‐reversal symmetry macroscopically, the magnetic octupole domains can be visualized using the magneto‐optical Kerr effects even though they only carry vanishingly small magnetization.^[^ ^25^ ^]^ The polarization of the magnetic octupole determines the distribution of Weyl points in the momentum space and, thus, the polarity of AHE. Recent developments in the thin film fabrication processes have led to the realization of various spintronic devices using the Mn_3_Sn film.^[^ ^26^, ^27^, ^28^, ^29^, ^30^, ^31^, ^32^, ^33^, ^34^ ^]^ In the antiferromagnetic memory made of the Mn_3_Sn/heavy metal multilayers, the electrical current may switch the direction of the ferroic order of the magnetic octupole (Figure 1b). Thus, the information is stored as the polarity of AHE, which may be read out through the sign and magnitude of the anomalous Hall voltage.

For application, it is highly important to develop technologies to enhance the readout signal. There are several ingredients that determine the magnitude, such as geometrical factors of a memory cell, shunting current, and volume fraction of the switching domain in the cell. While the former two are extrinsic and can be tuned artificially by changing the magnetic cell and layer structure, the last should be an intrinsic property of the cell made of a multilayer stack. To find a condition for the thin film to generate a large voltage signal, we prepared four different multilayer stacks: 1) Ru(2)/Mn_3_Sn(40)/Pt(5); 2) Mn_3_Sn(40)/Pt(5); 3) Ru(2)/Mn_3_Sn(40)/W(5); and 4) Mn_3_Sn(40)/W(5), all units in nm, deposited on a Si/SiO_2_ substrate (see Experimental Section). For samples 1)–3), the stacks are annealed at 450 °C for 0.5 h after the fabrication of the Mn_3_Sn layer. In addition to that, sample 4) Mn_3_Sn(40)/W(5) is annealed at 450 °C for 0.5 h after the fabrication of the W layer. The magnetic, transport, and structural properties of the Mn_3_Sn layer made by the same method have been reported previously in the previous studies.^[^ ^27^, ^34^ ^]^ Using the thin films, a Hall bar structure (16 μm × 96 μm) is fabricated, which is contacted with Ti/Au electrodes (Figure 1c). In our electrical switching experiment, both electrical current and bias field are applied along the longitudinal direction (*x*‐direction), and the transverse Hall voltage *V*_H_ is detected in the *y*‐direction. All transport measurements have been performed at room temperature. As shown in the schematic image for the SOT switching in Figure 1c, by taking Mn_3_Sn/W bilayer devices as an example, spin current generated by the spin Hall effect in the heavy metals exerts the SOT on the Mn_3_Sn spin texture and causes the switching of the polarization direction of the magnetic octupole.^[^ ^23^ ^]^

## 2 Results and Discussion

**Figure 2:**
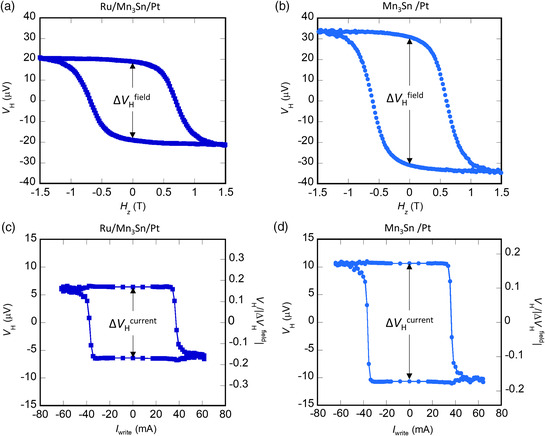
a,b) Hall voltage *V*_H_ as a function of perpendicular magnetic field *H*_*z*_ for Ru/Mn_3_Sn/Pt and Mn_3_Sn/Pt devices, respectively. c,d) *V*_H_ as a function of write current *I*_write_ with a bias field *μ*_0_ *H*_*x*_ = 0.1 T along the electrical current direction for Ru/Mn_3_Sn/Pt and Mn_3_Sn/Pt devices, respectively.

First, we compare the Hall voltages *V*_H_ obtained in both field and current switching processes in the multilayer stacks using Pt, i.e., the Ru/Mn_3_Sn/Pt and Mn_3_Sn/Pt heterostructures. **Figure** **2**a,b shows the Hall voltage *V*_H_ as a function of the out‐of‐plane magnetic field *H*_*z*_ measured under a read direct current (DC) of 0.2 mA. A clear hysteresis of *V*_H_ is observed in both types of samples and yields the zero‐field difference: Δ*V*_H_^field^ (=*V*_H_(+*H*_*z*_ → 0) − *V*_H_(−*H*_*z*_ → 0)). In the two types of the stack, a notable difference of Hall voltage is found; Δ*V*_H_^field^ ≈ 62 μV in Mn_3_Sn/Pt devices is ≈1.6 times larger than the one for the Ru/Mn_3_Sn/Pt devices.

On the other hand, the electrical switching is examined by sending a 100 ms write‐pulse current *I*_write_ along the *x*‐direction to drive SOT in Mn_3_Sn. To avoid heating effect, a wait time of 600 ms is inserted after turning off the pulse current and before measuring *V*_H_ with the read current of 0.2 mA (Figure S1, Supporting Information). Figure 2c,d shows *V*_H_ as a function of the write current *I*_write_ (*V*_H_ − *I*_write_ loop) with a bias field of *μ*_0_ *H*_*x*_ = 0.1 T along the current direction. We would like to note that in the field switching measurement, we applied a vertical shift to the raw data to diminish the offset in the Hall measurement. In current switching measurement, the same value for the vertical shift as the field switching is applied for the same device. For both Pt samples (with (w/) and without (w/o) Ru layer), a negative (positive) jump appears in the *V*_H_ − *I*_write_ loop under a positive (negative) current when *I*_write_ is larger than a critical write current *I*_c_. The magnitude of the jump, Δ*V*_H_^current^ for Mn_3_Sn/Pt devices, is found ≈21 μV, ≈1.6 times larger than the one in Ru/Mn_3_Sn/Pt devices, similar to the field sweep case. To check whether the measured Δ*V*_H_^current^ can come from the switching of any in‐plane components of the magnetic octupole of Mn_3_Sn, we measure *V*_H_ as the function of *H*_*x*_ (along the electrical current direction) and *H*_*y*_ (perpendicular to the current direction) in the Ru/Mn_3_Sn/Pt sample. We find that there is no notable difference in *V*_H_ between applying *H*_*x*_ and *H*_*y*_ (Figure S2a, Supporting Information). Thus, Δ*V*_H_^current^ must originate from the switching of the out‐of‐plane components of the magnetic octupole. As a result, the volume fraction of the domain switched perpendicularly can be estimated by taking the ratio Δ*V*_H_^current^/Δ*V*_H_^field^. This ratio is ≈33% in both types of Pt samples, consistent with the results reported previously.^[^ ^23^ ^]^ We also investigate if the current switching behavior can be affected by the different initial states of Mn_3_Sn, aligned to different out‐of‐plane directions, +*z* or −*z*. We find that the current switching loop does not depend on the initial state of Mn_3_Sn (Figure S2b, Supporting Information). The critical write current densities in the Pt layer, estimated by considering the current shunting effect, at both samples w/ and w/o Ru layer are ≈3 × 10^11^ A m^−2^, nearly the same as the previously reported values.^[^ ^23^ ^]^ This is of the same order of magnitude as or even smaller than those known for the switching devices using ferromagnets/heavy metal and antiferromagnets/heavy metal heterostructures.^[^ ^35^, ^36^, ^37^, ^38^, ^39^, ^40^, ^41^, ^42^ ^]^ We also notice that the shape of hysteresis in the current‐induced switching is sharper than the field‐induced one. This might be related to the difference in the switching mechanism. In the field‐induced case, the damped precession of magnetization related to the magnetic anisotropy energy may drive the switching and lead to the rounded shape of the hysteresis in the poly‐crystalline Mn_3_Sn. In contrast, the current‐induced one is more likely caused by the domain‐wall propagation, as generally observed in ferromagnets’ case. The domain‐wall switching might result in a sharper shape of the switching in the Hall voltage. In fact, the domain wall propagation by electrical current in a single‐crystalline bulk Mn_3_Sn has been reported recently.^[^ ^43^ ^]^ A similar mechanism should apply for our poly‐crystalline Mn_3_Sn thin film. A further study on the domain wall motion in Mn_3_Sn thin films may help explain the sharp shape of the electrical switching.

**Figure 3:**
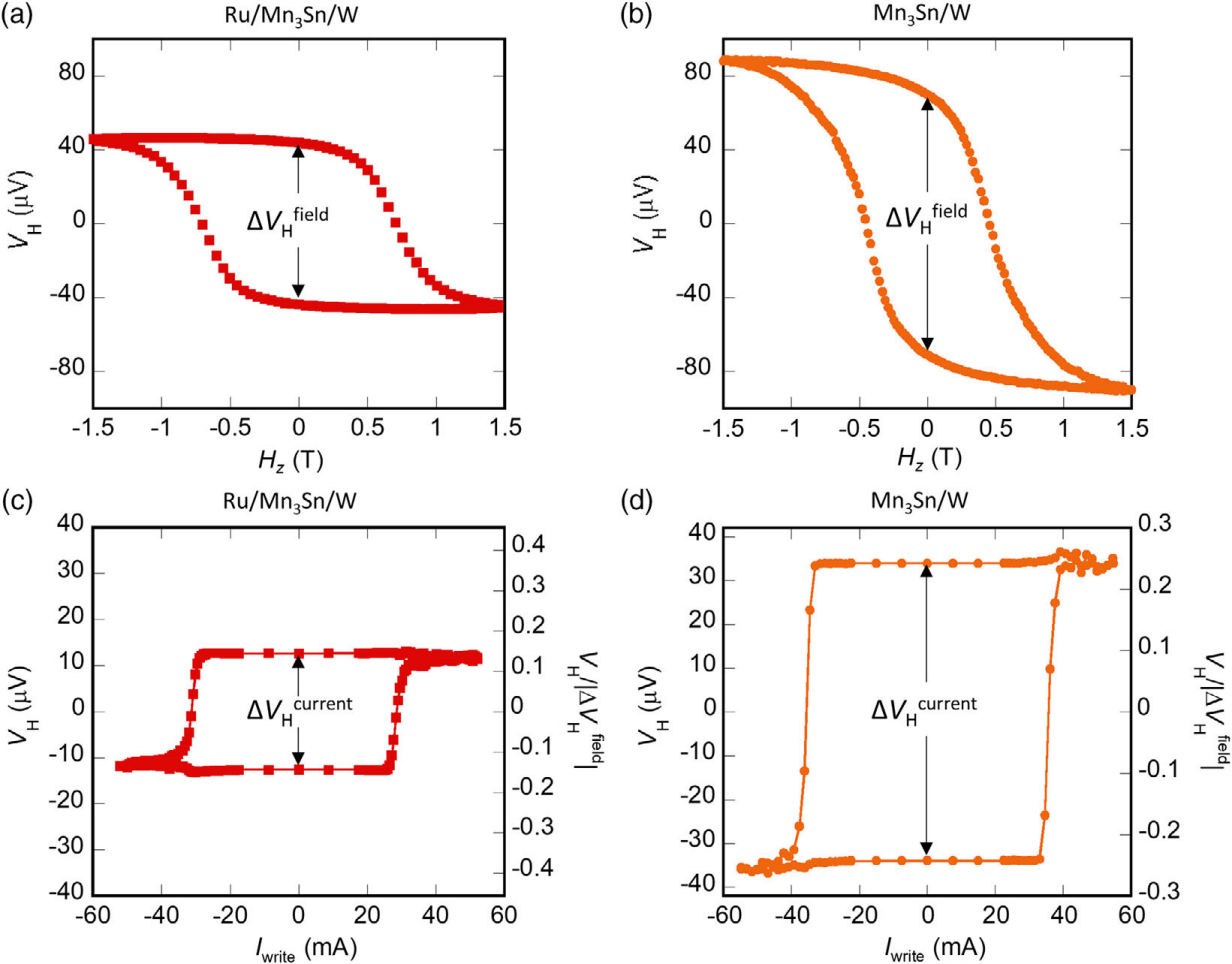
a,b) Hall voltage *V*_H_ as a function of perpendicular magnetic field *H*_*z*_ for Ru/Mn_3_Sn/W and Mn_3_Sn/W devices, respectively. c,d) *V*_H_ as a function of write current *I*_write_ under a bias field *μ*_0_ *H*_*x*_ = 0.1 T along the electrical current direction for Ru/Mn_3_Sn/W and Mn_3_Sn/W devices, respectively.

As in the Pt case mentioned earlier, the Hall voltage loops are also examined for the multilayer stacks using W in both field and current switching processes. First, the values of Δ*V*_H_^field^ of the W devices with and without Ru layer are found to be twice larger than the Pt devices (**Figure** **3**a,b). The materials dependence of heavy metals on AHE can be explained by the different resistivity and shunting effect of Pt and W layer. Calculated from the total resistance of devices measured by a two‐probe method, the resistivity of Ru/Mn_3_Sn, Mn_3_Sn, Pt, and W layer is estimated to be 280, 300, 45, and 160 μΩ cm, respectively, being consistent with our previous reports.^[^ ^23^, ^27^ ^]^ The shunting effect on the AHE can be calculated by ignoring the interface resistance (see Experimental Section). We find that the estimated Δ*V*_H_^field^ values of Ru(2)/Mn_3_Sn(40)/Pt(5) and Ru(2)/Mn_3_Sn(40)/W(5) are ≈33% and ≈68% of one in Ru(2)/Mn_3_Sn(40), respectively, and the estimated Δ*V*_H_^field^ values of Mn_3_Sn(40)/Pt(5) and Mn_3_Sn(40)/W(5) are ≈30% and ≈66% of the Mn_3_Sn(40) thin film, respectively. As a result, the value of Δ*V*_H_^field^ in the W devices is around twice as large as those in the Pt devices, being consistent with our experiment results. Second, in the current switching measurements (Figure 3c,d), the switching polarity of W devices is found opposite to the Pt devices. Namely, a positive (negative) jump of *V*_H_ appears under a positive (negative) current in a bias field *H*_*x*_. The difference in the polarity of the electrical switching between the Pt and W multilayers is consistent with the signs of the spin Hall angle of Pt (positive) and W (negative), demonstrating the SOT mechanism in the magnetic switching.^[^ ^23^ ^]^

Next, let us compare the W devices with and without the Ru layer. For the field switching, Δ*V*_H_^field^ of ≈140 μV in Mn_3_Sn/W devices is ≈1.6 times larger than the one with the Ru layer. This enhancement is the same as the case of Mn_3_Sn/Pt and Ru/Mn_3_Sn/Pt devices. In contrast, a significant enhancement in the ratio Δ*V*_H_^current^/Δ*V*_H_^field^ is found in Mn_3_Sn/W devices by removing the Ru layer, which is different with the Pt case. In the device w/o the Ru layer, a strikingly large Δ*V*_H_^current^ ≈ 70 μV is found, leading to a large ratio of Δ*V*_H_^current^/Δ*V*_H_^field^ ≈ 50%, whereas the insertion of Ru suppresses the ratio down to ≈29% for the Ru/Mn_3_Sn/W device (Δ*V*_H_^current^ ≈ 25 μV). On the other hand, we find that only in the Mn_3_Sn/W device, the zero‐field Hall voltage is less than the *V*_H_ value obtained at 1.5 T (Figure 3b). Further measurements of the field dependence of *V*_H_ up to 5 T reveal that 1.5 T is enough to obtain the maximum value of *V*_H_, because it is almost the same as the saturated value obtained at 2 T (Figure S6, Supporting Information). To examine if the enhanced Δ*V*_H_^current^/Δ*V*_H_^field^ does not originate from the decrease in the zero‐field Hall voltage, we calculate the ratio between Δ*V*_H_^current^ and Δ*V*_H_^field^ at 1.5 T of all the Pt and W samples. In particular, Δ*V*_H_^current^/Δ*V*_H_^field = 1.5 T^ of Mn_3_Sn/W devices turns out to be 37%, which is still 1.3 times larger than all other samples. A slight enhancement of the critical current density is found in w/o Ru layer samples, which are 7 × 10^10^ and 8 × 10^10^ A m^−2^ for the W sample w/ and w/o Ru layer, respectively.

Now, let us scrutinize the heterostructure dependence of the readout signals in the Pt and W devices to find the mechanism behind their enhancement. Our results indicate that in both Pt and W devices, the removal of the Ru layer leads to ≈60% enhancement in the switching Hall voltage Δ*V*_H_^field^ obtained in the field cycle. The first plausible reason for this is that by suppressing the shunting effect of the Ru layer, more current flows in Mn_3_Sn itself and results in the larger Hall voltage Δ*V*_H_^field^. Using the resistance of the Ru/Mn_3_Sn devices and the reported resistivity, 300 μΩ cm, of Mn_3_Sn(40) thin films,^[^ ^27^ ^]^ the resistivity of the Ru layer is calculated to be ≈120 μΩ cm, resulting in a 6% and 9% current shunting effect in Ru/Mn_3_Sn/Pt and Ru/Mn_3_Sn/W samples, respectively. By a further calculation considering an equivalent circuit of AHE (see Experimental Section), removing the Ru layer in Ru/Mn_3_Sn/Pt and Ru/Mn_3_Sn/W devices contributes to ≈14% and ≈20% enhancement of Δ*V*_H_^field^, respectively, and by itself does not explain the observed enhancement by ≈60%.

Second, the crystal orientation of the Mn_3_Sn grains may play an important role in the enhancement. In the Hall effect measurement, only the Mn_3_Sn grains that have the out‐of‐plane component of the magnetic octupole polarization contribute to the Hall voltage,^[^ ^9^ ^]^ and hereafter, we call such grains to be “AHE‐relevant.” However, the insertion of the Ru buffer layer renders anisotropic distribution of the Mn_3_Sn crystal orientation; the Mn_3_Sn grain prefers having the kagome plane and, thus, the octupole polarization parallel to the substrate surface.^[^ ^31^, ^34^ ^]^ The previous report finds that this anisotropic feature extends along the thickness direction up to 20 nm from the Ru layer and weakens with further increasing the distance.^[^ ^31^ ^]^ Thus, the Mn_3_Sn grains far from the Ru layer have a more random orientation of the kagome plane and produce larger AHE than those located near the Ru layer. In contrast, the polycrystalline grains in the thin film deposited directly on the Si/SiO_2_ substrate most likely have random crystal orientation for each grain irrespective of its distance from the substrate,^[^ ^27^ ^]^ thus resulting in more numbers of AHE‐relevant grains producing larger AHE than the one with the Ru layer. Therefore, the large electrical switching voltage found in the Mn_3_Sn/Pt device w/o Ru layer should mainly come from Δ*V*_H_^field^ enhanced due to crystal orientation.

**Figure 4:**
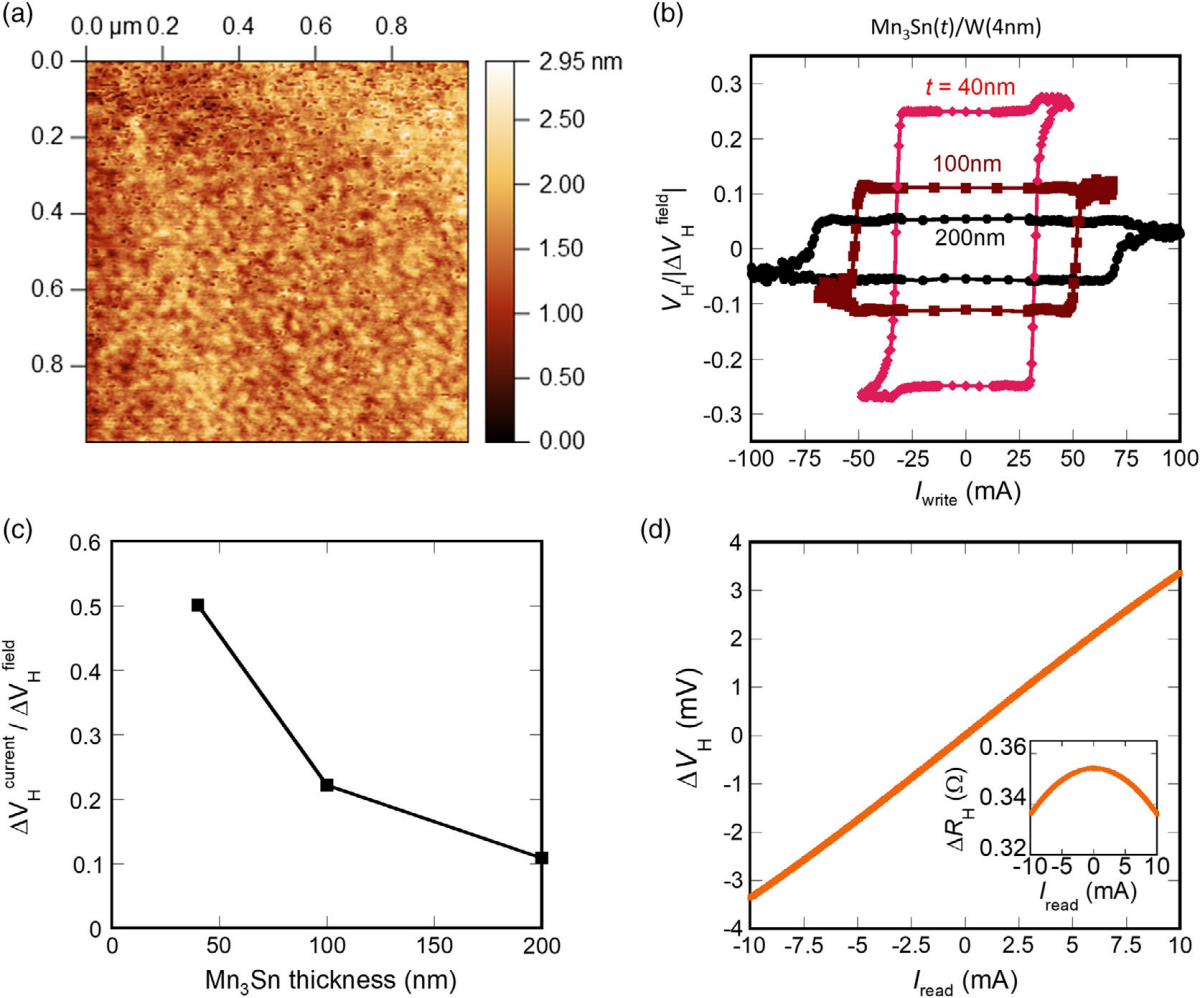
a) AFM image of a Mn_3_Sn(40)/W(5) Hall bar device. The RMS roughness is ≈0.5 nm. b) Δ*V*_H_^current^/|Δ*V*_H_^field^| as a function of write current *I*_write_ under a bias field of *μ*_0_ *H*_*x*_ = 0.07 T along the electrical current direction for Mn_3_Sn(40,100,200)/W(4) devices. c) Mn_3_Sn thickness dependence of Δ*V*_H_^current^/Δ*V*_H_^field^ in Mn_3_Sn(40,100,200)/W(4) samples. d) Switching Hall voltage Δ*V*_H_ as a function of the read current *I*_read_ in Mn_3_Sn(40)/W(5) sample. Inset: Hall resistance Δ*R*_H_ as a function of the read current *I*_read_.

For Ru/Mn_3_Sn/Pt and Mn_3_Sn/Pt devices, the ratios of Δ*V*_H_^current^/Δ*V*_H_^field^ observed are both ≈33%. In contrast, Mn_3_Sn/W heterostructures show a large difference in Δ*V*_H_^current^/Δ*V*_H_^field^, depending on the existence/absence of the Ru buffer layer. Mn_3_Sn/W devices have the ratio of ≈50%, whereas it is ≈29% in Ru/Mn_3_Sn/W devices. This difference between the Pt and W devices is possibly related to the annealing process taken after the deposition of the Mn_3_Sn/W thin film, which is absent in the fabrication process of the Pt devices. The Mn_3_Sn layer in all devices is once annealed at 450 °C for 0.5 h after its deposition at room temperature. Therefore, the structural properties of the Mn_3_Sn layer would not change even if an additional annealing process takes place later. In fact, the AHEs in the field dependence for Mn_3_Sn/Pt and Mn_3_Sn/W are both ≈1.6 times larger than those with the Ru seed layer, confirming that their crystal has very similar quality. On the other hand, the additional annealing process may lead to the atomic rearrangement at the interface between the W and Mn_3_Sn layers. If this is the case, the enhancement in Δ*V*_H_^current^/Δ*V*_H_^field^ is likely due to the interface condition unique to the Mn_3_Sn/W interface. To examine the interface condition in Mn_3_Sn/W and Ru/Mn_3_Sn/W thin films, we make the atomic force microscope (AFM) measurements to estimate the total roughness of the thin films. **Figure** **4**a shows the AFM image of one Mn_3_Sn/W device. The root mean square (RMS) roughness of Mn_3_Sn/W is found to be ≈0.5 nm, which is one order smaller than the case (≈few nm) of Ru/Mn_3_Sn/W films.

In addition, to check if there is any reaction between materials after the annealing process, we perform the X‐ray diffraction measurements on Mn_3_Sn/W thin films (Figure S4, Supporting Information). We find that there could be a Mn_2_WSn mixing layer formed at the interface, consistent with the report made by Yoon et al.^[^ ^31^ ^]^ The reported Curie temperature of Mn_2_WSn is ≈260 K,^[^ ^44^ ^]^ so it may not contribute to the AHE signal under zero perpendicular field at room temperature. To understand the possible influence of a ferromagnetic Mn_2_WSn layer on AHE, we investigate the field dependence of the Hall resistivity of the Mn_3_Sn/W sample from 200 to 300 K (Figure S6, Supporting Information). Both the magnetization and AHE signal of Mn_2_WSn become one order of magnitude larger at 200 K than 300 K.^[^ ^44^ ^]^ In contrast, the total Hall resistivity of the Mn_3_Sn/W thin film at 200 K is slightly smaller than the one at 300 K. This suggests that the AHE contribution from Mn_2_WSn is negligibly small compared with the one from Mn_3_Sn. In addition, as there is no notable decrease but only enhancement in AHE signal of Mn_3_Sn/W samples compared with the one with Ru layer, the Mn_2_WSn mixing layer should appear only in a very narrow region near the interface, and the values of Δ*V*_H_^current^/Δ*V*_H_^field^ come only from the switching of AHE in the Mn_3_Sn layer itself. Our results confirm that the interface condition controls Δ*V*_H_^current^/Δ*V*_H_^field^; decreasing the interface roughness and/or the formation of Mn_2_WSn mixing layer could enhance the readout signal. On the other hand, we find that the annealing process for Mn_3_Sn/Pt samples leads to the reaction between Pt and the whole Mn_3_Sn layer. Therefore, the annealing method is not suitable for Pt devices (Figure S4 and S5, Supporting Information).

Further comparison between the Δ*V*_H_^current^/Δ*V*_H_^field^ ratios measured using various Mn_3_Sn/heavy metals heterostructures provides the information of the switching mechanism in Mn_3_Sn. First, the same Δ*V*_H_^current^/Δ*V*_H_^field^ ratio ≈33% found in both Pt devices w/ and w/o Ru layer clarifies that the switching does occur not only in a narrow region approximately few nm near the Mn_3_Sn/Pt interface but also in the AHE‐relevant crystal grains in the entire part of the 40 nm Mn_3_Sn layer. As discussed earlier, in the device w/ Ru, the Mn_3_Sn grains near the Ru layer align their kagome planes nearly parallel to the substrate and, thus, should contribute to the AHE signal much less than those near heavy metal and far from the Ru layer. In contrast, the heterostructure w/o the Ru layer should have a random distribution of the crystal grain orientation over the entire Mn_3_Sn layer and, thus, produce AHE homogeneously. Therefore, suppose the switching of the magnetic octupole occurred only in the AHE‐relevant grains in a narrow region, for example, 33% of 40 nm, 13 nm thickness near the Mn_3_Sn/Pt interface, a notable difference in Δ*V*_H_^current^/Δ*V*_H_^field^ would have been seen between the two devices w/ and w/o the Ru layer. However, it is not the case in our experiment. Thus, the observed value of ≈33% should originate from the magnetic octupole switching of the AHE‐relevant Mn_3_Sn grains in the entire layer; a 40 nm depth of the Mn_3_Sn layer may be switched electrically through the SOT.

To obtain further information on effective switching thickness, we investigate the switching phenomena by changing the thickness of the Mn_3_Sn layer in the W heterostructure. In particular, using three types of the Hall bar devices: Mn_3_Sn((v) 40, (vi) 100, (vii) 200)/W(4) (see Experimental Section), we perform the field and electrical switching measurements (Figure 4b) and estimate their Δ*V*_H_^current^/Δ*V*_H_^field^ by the same protocol as mentioned earlier (Figure 4c). For Mn_3_Sn(100, 200)/W(4) samples, we find that the offset of the measured Hall voltage in the current switching process depends on the octupole direction of the initial states, which is not observed in Mn_3_Sn(40) samples (Figure S3, Supporting Information). Thus, only in Figure 4b, the offset is subtracted by centering the switching loop. We find a clear decrease in Δ*V*_H_^current^/Δ*V*_H_^field^ with increasing the thickness from 40 to 100 and 200 nm. Given the identical fabrication process utilized for all the devices, the interface between Mn_3_Sn and W should be at the same condition. In fact, we confirm that all have the RMS roughness of ≈0.5 nm. It is reasonable to consider that the switching volume fraction at the interface is also the same. Thus, the decreasing Δ*V*_H_^current^/Δ*V*_H_^field^ as a function of thickness indicates that the switching involves a limited depth of Mn_3_Sn near the heavy metal, smaller than the total thickness. Assuming that a certain Mn_3_Sn region adjacent to the interface has the same switching ratio Δ*V*_H_^current^/Δ*V*_H_^field^ as the one (≈50%) observed in the 40 nm device, we may estimate the depth of the corresponding region next to the W film to be 44 and 43 nm in the 100 and 200 nm Mn_3_Sn samples, respectively. This suggests that the active Mn_3_Sn layer for the electrical switching has the maximum thickness of ≈40 nm, which is much thicker than that of conventional ferromagnetic and ferrimagnetic materials.^[^ ^35^, ^36^, ^45^, ^46^, ^47^ ^]^ The spin diffusion length reported for Mn_3_Sn is only ≈1 nm and is much shorter than our estimation of 40 nm for the switching thickness.^[^ ^34^ ^]^ If there is no domain wall in the 40 nm thickness, the magnetic domain reversal should be determined by the injected total spin angular momentum and not limited by the spin diffusion length. Thus, the estimated 40 nm might be related to the size of magnetic domain of Mn_3_Sn in the out‐of‐plane direction. A future study on the thickness dependence of Mn_3_Sn film thinner than 40 nm in the switching measurement is required to clarify that whether the SOT switching can occur in a 40 nm thickness of Mn_3_Sn.

Our experimental results indicate that the Hall voltage signal due to the electrical switching can be enhanced: 1) by removing the Ru buffer layer and 2) by making the smooth interface and/or forming the Mn_2_WSn mixing layer by annealing. The combination of the enhancement in both Δ*V*_H_^field^ and Δ*V*_H_^current^/Δ*V*_H_^field^ results in the largest switching Hall voltage of ≈70 μV and a Hall resistance of ≈0.35 Ω observed in the Mn_3_Sn/W devices, reaching one order of magnitude larger value than the previous report for the Ru/Mn_3_Sn/Pt device.^[^ ^23^ ^]^ The switching Hall voltage should be further enhanced by applying larger read current if the temperature of devices is kept in a suitable range. To achieve the maximum switching signal, we investigate the readout Hall voltage *V*_H_ and Hall resistance *R*_H_ as a function of the electrical current using the Mn_3_Sn/W devices (Figure 4d). We monitor if *R*_H_ decreases as *I*_read_ increases, following the temperature dependence of AHE, as reported previously.^[^ ^9^, ^27^ ^]^ A large Hall voltage of ≈1 mV is obtained with *I*_read_ = 3 mA, whereas the difference between *R*_H(*I*=3 mA)_ and *R*_H(*I*=0.2 mA)_ is only 0.4%. This resistance change is the same order as the noise level, and thus, the heating effect can be neglected.

Recently, antiferromagnets (AFs) have attracted tremendous interest as next generation active material for spintronics for designing a high‐density and ultrafast speed device, as they may have no stray fields perturbing the neighboring cells and have much faster dynamics of the order of picosecond than the ferromagnetic counterparts (nanosecond).^[^ ^48^, ^49^ ^]^ It has only been a few years since the first demonstration was made for the electrical switching of an antiferromagnetic domain of collinear AFs.^[^ ^38^, ^39^, ^40^, ^41^, ^42^, ^50^, ^51^, ^52^ ^]^ Because these AFs do not break the macroscopic time‐reversal symmetry, AHE cannot be used for detecting the magnetic switching. Instead, anisotropic magnetoresistance (AMR) is used. For the DC detection of the AMR switching signal, one needs to apply write/read current and measure the Hall voltage at four different directions.^[^ ^38^, ^39^, ^40^, ^41^, ^42^, ^50^, ^51^, ^52^ ^]^ This is twice of the normal Hall measurement setup where only two direction perpendicular to each other is required for applying current and reading voltage. On the other hand, the alternative current measurement enables the detection of AMR switching using a normal Hall measurement configuration.^[^ ^51^ ^]^ However, all these methods are more complicated than the DC switching measurement performed in ferromagnets. In contrast, our device based on the antiferromagnetic Weyl semimetal Mn_3_Sn is much simpler, requiring only normal Hall measurement setup and its DC detection, and, thus, can be fabricated/operated with the same protocols as those used for the conventional spintronics devices based on ferromagnets. In contrast, our device based on the antiferromagnetic Weyl semimetal Mn_3_Sn is much simpler, requiring only normal DC Hall measurement setup, and, thus, can be fabricated/operated with the same protocols as those used for the conventional spintronics devices based on ferromagnets. While the magnitude is still smaller than those found in magnetic tunneling junctions using ferromagnets, the observed mV order Hall voltage provides a significant impact on the electrical switching technology. In particular, the binary signals with different signs may completely suppress the reading errors, as they do not overlap with each other. Thus, it paves the path for designing a new type of memory device by taking advantage of the topological robust properties of Weyl antiferromagnets.

## 3 Experimental Section

3.1

3.1.1

##### Sample Preparation

In this study, we prepared seven different multilayer stacks: 1) Ru(2)/Mn_3_Sn(40)/Pt(5); 2) Mn_3_Sn(40)/Pt(5); 3) Ru(2)/Mn_3_Sn(40)/W(5); 4) Mn_3_Sn(40)/W(5); 5) Mn_3_Sn(40)/W(4); 6) Mn_3_Sn(100)/W(4); and 7) Mn_3_Sn(200)/W(4), all units in nm, deposited on a Si/SiO_2_ substrate. All films have a 2–3 nm AlO_*x*_ capping layer to prevent the oxidation. The Mn_3_Sn, Ru, and Pt layers are deposited at room temperature using a DC magnetron sputtering system with a base pressure of less than 5 × 10^−7^ Pa. The W layer is fabricated at room temperature by molecular beam epitaxy (MBE) under ultrahigh vacuum with a base pressure of less than 2 × 10^−8^ Pa. All the fabrication processes are in situ conditions including the sample transfer from the sputter chamber to the molecular beam epitaxy chamber. For samples 1)–3), the stacks are annealed at 450 °C for 0.5 h after the fabrication of the Mn_3_Sn layer and then fabricated the Pt or W layer. For samples 4)–7), Mn_3_Sn/W is annealed at 450 °C for 0.5 h after the fabrication of the W layer. The composition of the Mn_3_Sn layer is Mn_3.04(2)_Sn_0.96(2)_, as determined by scanning electron microscopy energy‐dispersive X‐ray spectrometry (SEM‐EDX).

##### Shunting Effect on the AHE in Mn_3_Sn/NM Bilayer

The AHE in the multilayer made by ferromagnets and nonmagnetic (NM) materials, such as Fe/Cu bilayers and Pd/Co/Pd trilayers,^[^ ^53^, ^54^ ^]^ has been analyzed by the shunting effect and the equivalent circuit of AHE. Here, by ignoring the interface resistance, we use a simple model to calculate the shunting effect of the NM layer on AHE in Mn_3_Sn/NM bilayers.^[^ ^53^, ^54^ ^]^ First, for the current flowing in the *x*‐direction (longitudinal direction), the Mn_3_Sn and NM layers can be considered as two resistors connected in parallel. The current flowing along the *x*‐direction in the Mn_3_Sn layer, $I_{x}^{\text{Mn}3\text{Sn}}$, is described as

(1) $$I_{x}^{\text{Mn}3\text{Sn}}=I_{x}\frac{R_{\text{NM}}}{R_{\text{Mn3Sn}}+R_{\text{NM}}}=I_{x}{\left(1+\frac{\rho_{\text{Mn3Sn}}}{\rho_{\text{NM}}}\frac{t_{\text{NM}}}{t_{\text{Mn3Sn}}}\right)}^{-1}$$

where $I_{x}$ is the total current flowing in the bilayer samples. $\rho_{\text{Mn3Sn}}$, $\rho_{\text{NM}}$, $t_{\text{Mn}3\text{Sn}}$, and $t_{\text{NM}}$ are the resistivity of Mn_3_Sn, resistivity of NM, thickness of Mn_3_Sn, and thickness of NM, respectively.

Second, the Hall resistivity of a single Mn_3_Sn film is defined by $\rho_{xy}^{\text{Mn}3\text{Sn}}=t_{\text{Mn3Sn}}V_{y}^{\text{Mn}3\text{Sn}}/I_{x}^{\text{Mn}3\text{Sn}}$, where $\rho_{xy}^{\text{Mn}3\text{Sn}}$ and $V_{y}^{\text{Mn}3\text{Sn}}$ are the Hall resistivity and the measured Hall voltage of the Mn_3_Sn thin film, respectively. When an NM layer contacts with the Mn_3_Sn layer, the AHE potential from Mn_3_Sn drives a transverse current in the NM layer. Thus, for the transverse direction, the bilayer can be considered as a closed circuit of two resistors connected in series with electrical potential $V_{y}^{\text{Mn}3\text{Sn}}$. The total Hall voltage in the bilayer can be calculated by

(2) $$V_{y}=V_{y}^{\text{Mn}3\text{Sn}}\frac{R_{\text{NM}}}{R_{\text{Mn3Sn}}+R_{\text{NM}}}=V_{y}^{\text{Mn}3\text{Sn}}{\left(1+\frac{\rho_{\text{Mn3Sn}}}{\rho_{\text{NM}}}\frac{t_{\text{NM}}}{t_{\text{Mn3Sn}}}\right)}^{-1}$$

Substituting Equation (1) and (2) into $\rho_{xy}^{\text{Mn}3\text{Sn}}$ yields

(3) $$\rho_{xy}^{\text{Mn}3\text{Sn}}=t_{\text{Mn3Sn}}\frac{V_{y}}{I_{x}}{\left(1+\frac{\rho_{\text{Mn3Sn}}}{\rho_{\text{NM}}}\frac{t_{\text{NM}}}{t_{\text{Mn3Sn}}}\right)}^{2}$$

Assuming that the Hall resistivity of the Mn_3_Sn film does not depend on the NM layer, we can estimate the total Hall voltage from the resistivity and thickness of the Mn_3_Sn and NM layers.

Calculating from the total resistance of devices measured by a two‐probe method, the estimated resistivities of the Ru/Mn_3_Sn, Mn_3_Sn, Pt, and W layers are 280, 300, 45, and 160 μΩ cm, respectively. Using these parameters, the values of Δ*V*_H_^field^ of Ru(2)/Mn_3_Sn(40)/Pt(5) and Ru(2)/Mn_3_Sn(40)/W(5) are estimated to be ≈33% and ≈68% of one in Ru(2)/Mn_3_Sn(40), respectively; the values of Δ*V*_H_^field^ of Mn_3_Sn(40)/Pt(5) and Mn_3_Sn(40)/W(5) are estimated to be ≈30% and ≈66% of Mn_3_Sn(40), respectively.

In the calculation of the shunting effect of Ru layer in Ru/Mn_3_Sn/Pt or W, we use the estimated resistivity of 120 μΩ cm for Ru, which is calculated from the total resistance of Ru(2)/Mn_3_Sn(40) and the reported resistivity, 300 μΩ cm, of 40 nm Mn_3_Sn thin film.^[^ ^27^ ^]^ Considering the Mn_3_Sn(40)/Pt or W(5) as a 45 nm layer, removing the Ru layer from Ru/Mn_3_Sn/Pt and Ru/Mn_3_Sn/W devices contributes to ≈14% and ≈20% enhancement of Δ*V*_H_^field^, respectively.

## Conflict of Interest

The authors declare no conflict of interest.

## Data Availability Statement

The data that support the findings of this study are available from the corresponding author upon reasonable request.

## Supporting information

Supplementary Material
