# Supplementary material for: Large Hall Signal due to Electrical Switching of an Antiferromagnetic Weyl Semimetal State
Source: Small Sci. 2021 Apr 15;1(5):2000025. doi: 10.1002/smsc.202000025 (PMC11935791; doi:10.1002/smsc.202000025)
Supplement: Supplementary file 1 — Supplementary Material [file SMSC-1-2000025-s001.pdf]

Supporting Information

Large Hall signal due to electrical switching of an antiferromagnetic Weyl semimetal state

Hanshen Tsai, Tomoya Higo, Kouta Kondou, Shoya Sakamoto, Ayuko Kobayashi, Takumi Matsuo, Shinji Miwa, Yoshichika Otani, and Satoru Nakatsuji*

Wait time dependence of current switching measurement

We perform the current switching measurement in the Ru/Mn_3Sn/Pt sample with a few different waiting times between write and read current from 600ms to 5s as shown in Fig. S1. There is no notable difference found between different wait times. This is consistent with the results reported in Ref. [23] that the waiting time of 600 ms after the write current is long enough to cool down the sample to the room temperature.

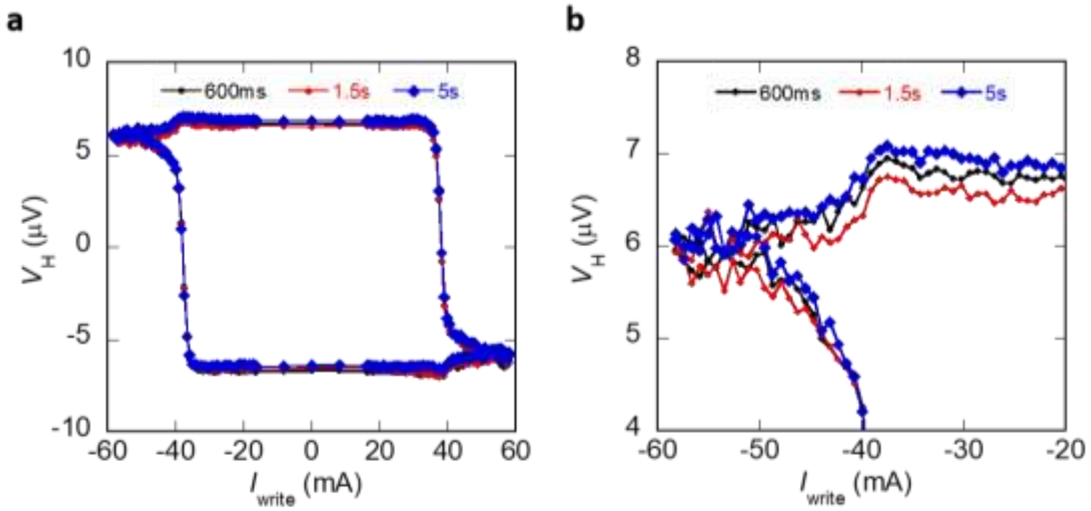

Figure S1. (a) V_H as a function of the write-current I_{write} with different wait time between write and read operations. A bias field H_x = 0.1 T is applied in the whole measurement. (b) Enlarged version of Fig. S1a.

Hall voltage measured in different field directions in Ru/Mn_3Sn/Pt

To understand whether the Hall voltage measured in our devices can come from the in-plane component of the magnetic octopule of Mn_3Sn, we investigate the field dependence of the Hall voltage under fields along the *z*-direction (out-of-plane direction), the *x*-direction (along the current direction), and the *y*-direction (perpendicular to the current direction) (Figure S2a). No clear difference of the Hall voltage is found for Mn_3Sn under fields aligned to the *x*- and *y*-directions. These results indicate that the switching Hall voltage does not come from the switching of the in-plane component but the out-of-plane components of the magnetic octopule.

Current switching measurement with different initial states in Ru/Mn_3Sn(40)/Pt and Mn_3Sn(40, 100, 200)/W

For the current switching measurements shown in Figures 2 and 3 in the main text, the alignment of the magnetic octupole of Mn_3Sn is not set to a certain initial direction by a magnetic field. Instead, we first applied a write current larger than critical current in a bias field H_x = 0.1 T to set the initial state, and then sweep the write current from positive to negative and vice versa to complete one loop. We repeat the current sweep to make a few loops and make the final measurements after checking the reproducibility.

To understand if the current switching behaviors can be changed with different initial states of Mn_3Sn, we apply the magnetic field along the z-diretion to set the initial state of Mn_3Sn in Ru(2)/Mn_3Sn(40)/Pt(5) samples and subsequently start the current switching measurement. The result is shown in Figure S2b. Red(blue) points correspond to an initial state aligned to positive(negative) out-of-plane direction by applying +1.5T(-1.5T) along the *z*-direction. We first increase the write current from 0 to a positive value, and then sweep from positive to negative and vice versa. We find that the initial state with+*z* or -*z* is not changed

under a current smaller than the critical current. When the write current exceedes the critical current, both +*z* and -*z* states are switched to the same state with the same Hall voltage. In addition, the magnitude of critical current and the shape of current switching loops do not depend on the initial states of Mn_3Sn.

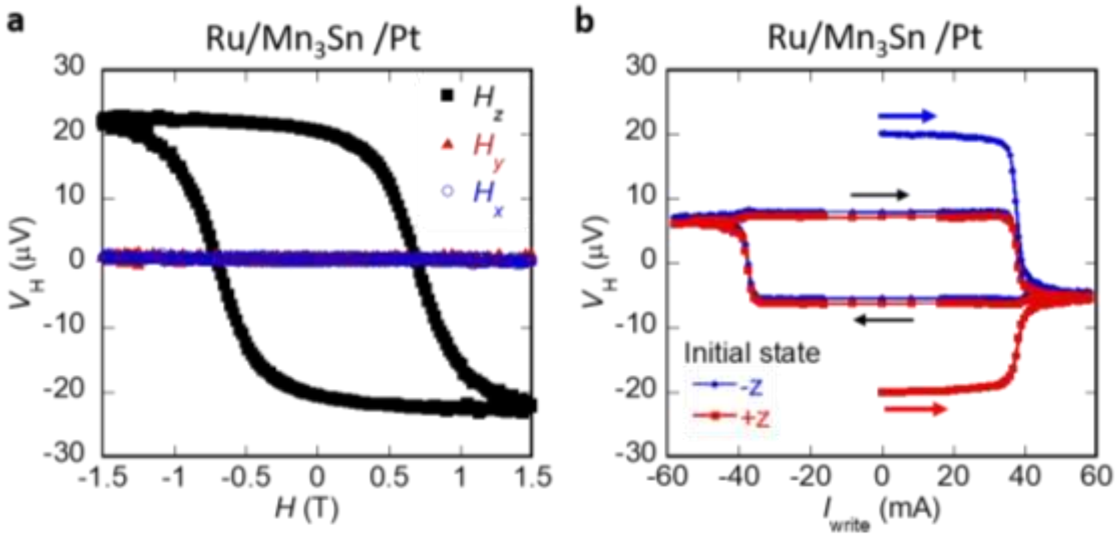

Figure S2 (a) Hall voltage of Ru/Mn_3Sn/Pt sample vs. the magnetic field applied along the *z*-direction (out-of-plane direction), the *x*-direction (along the electrical current direction), and the *y*-direction (perpendicular to the current direction). (b) The current switching of the Hall voltage in Ru(2 nm)/Mn_3Sn(40 nm)/Pt(5 nm) sample starting from different initial states of Mn_3Sn at room temperature. Red (blue) curve shows the current switching after applying a +1.5T(-1.5T) magnetic field along the *z*-direction. A 0.1T bias field is applied along the curretn direction in whole switching measruement.

We further perform the current switching measurements in Mn_3Sn(40, 100, 200)/W with different initial states, +1.5T H_z, -1.5T H_z, and +1.5T H_y (Fig. S3). We confirmed the history dependence in the virgin curve in all the samples with different thickness. Irrespective of the initial states, the application of large enough electrical current leads to the same state with a small Hall voltage, which becomes suppressed with increasing thickness. In addition, we found that in the thick Mn_3Sn samples, the switching loops are slightly shifted from the center

of AHE (Fig. S3), depending on the initial states. The magnitude of shift from the center differs for different thickness, ~3% and ~6% of the total AHE signal in Mn_3Sn(100)/W and Mn_3Sn(200)/W samples, respectively. These shifts indicate that there are some Mn_3Sn domains which cannot be switched by the SOT but remain their small out-of-plane components of magnetization during the current switching loops

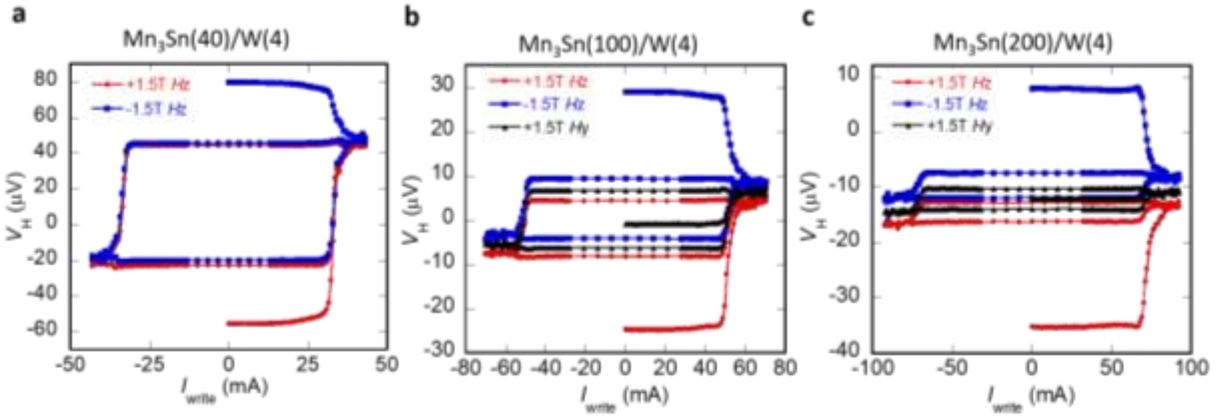

Figure S3. Hall voltage vs. write current loops for the current switching in (a) Mn_3Sn(40)/W(5), (b) Mn_3Sn(100)/W(5), (c) Mn_3Sn(200)/W(5) samples, starting from different initial states. The red, blue, and black curves show the current switching after applying +1.5 T H_z, -1.5 T H_z, and +1.5 H_y magnetic field before starting the measurements, respectively.

X-ray diffraction (XRD) analysis of Mn_3Sn(40)/Pt, W(5) thin film made with annealing after Pt, W deposition

The XRD patterns of Mn_3Sn/Pt or W fabricated with annealing process after Pt or W deposition are shown in Fig. S4. In Mn_3Sn/W sample, most of the peaks match well with those for the poly-Mn_3Sn thin film reported in Ref. [27] except a broad peak around 40 degree. This broad peak should come from either Mn_3Sn(002), Mn_2WSn(002), or W(011), indicating the possible existence of Mn_2WSn impurity as reported in Ref. [31]. On the other hand, no peaks of poly-Mn_3Sn appear in the Mn_3Sn/Pt sample obtained after annealing for the

preparation of the interface, except one large and broad peak at 40 degree. The total absence of other Mn_3Sn peaks is most likely due to the reaction between Pt and the whole Mn_3Sn layer, leading to the formation of Mn_2PtSn layer.

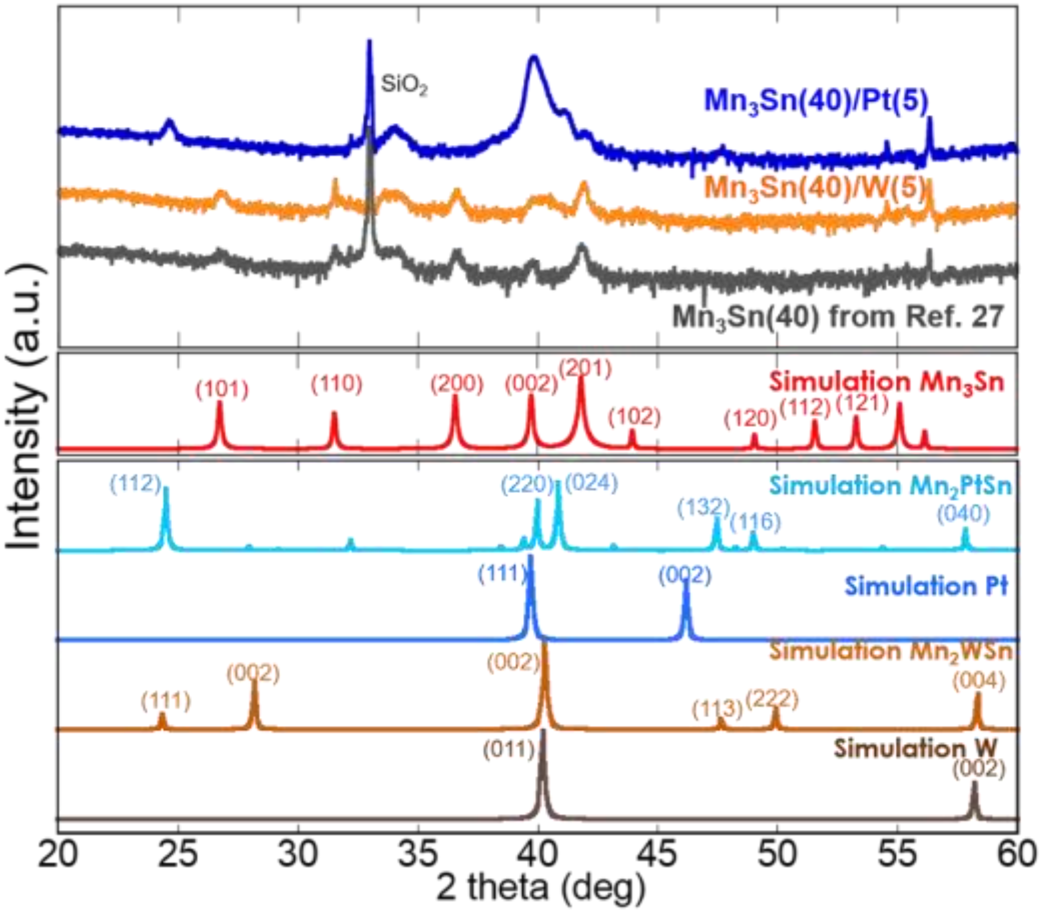

Figure S4. X-ray diffraction spectra (Cu-Kα) for Mn_3Sn(40)/Pt(5) and Mn_3Sn(40)/W(5) obtained with annealing process after Pt or W deposition. Result of Mn_3Sn(40) is adapted from Ref. 27. Simulated spectra for Mn_3Sn, Mn_2PtSn, Pt, Mn_2WSn, and W are also shown.

Hall effect in Mn_3Sn(40)/Pt(5) thin film obtained with annealing process after Pt deposition

In the XRD pattern of Mn_3Sn(40)/Pt(5) fabricted with annealing process after Pt deposition (Fig. S4), there is a large peak observed at 40 degree which may consist of Mn_2PtSn or Mn_3Sn(002). To further confirm the absence of Mn_3Sn phase in the film, we investigate the Hall effect of this sample at room temperature (Fig. S5). Clearly, the sign of AHE is opposite

to the one for Mn_3Sn and there is no coercivity observed, indicating that the phase formed in the film is not Mn_3Sn but other compounds such as Mn_2PtSn or MnPtSn. Thus, the annealing method for enhancing the switching signal is not suitable for Mn_3Sn/Pt sample.

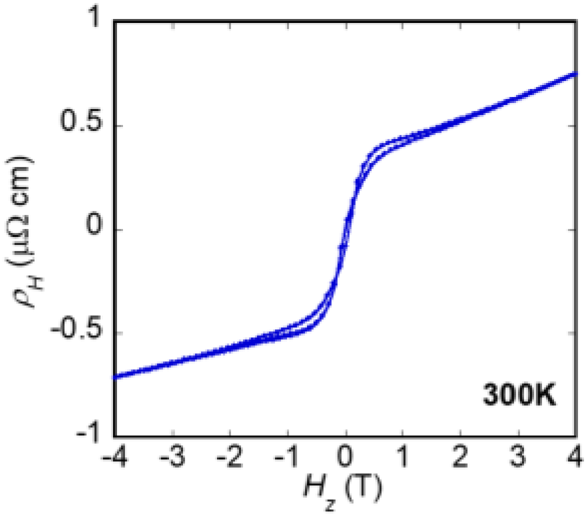

Figure S5. Hall resistivity as a function of out-of-plane magnetic field H_z in $Mn_3Sn(40)/Pt(5)$ obtained with annealing process after Pt deposition.

Anomalous Hall resistivity of Mn_3Sn/W from 200K to 300K

The anomalous Hall resitance R_H of Mn_3Sn/W sample is measured at 200 K, 240 K, and 300 K under field up to 5T (Fig. S6). We found that the 1.5 T field used in this study is large enough to obtain the maximum value of R_H since it is almost the same as the saturated value obtained at 2 T.

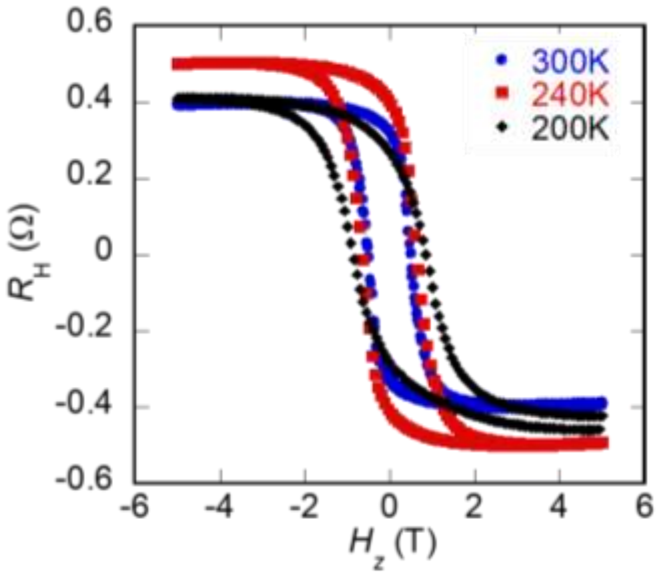

Figure S6. Hall resistance R_H of Mn_3Sn/W sample vs. the out-of-plane magnetic field H_z at 200 K, 240 K and 300 K.
